# Comment on Lunz et al. Impact and Modification of the New PJI-TNM Classification for Periprosthetic Joint Infections. *J. Clin. Med.* 2023, *12*, 1262

**DOI:** 10.3390/jcm12186073

**Published:** 2023-09-20

**Authors:** Volker Alt, Nike Walter, Markus Rupp, Susanne Baertl

**Affiliations:** 1 Department for Trauma Surgery, University Hospital, 93053 Regensburg, Germany; nike.walter@ukr.de (N.W.); markus.rupp@ukr.de (M.R.); susanne.baertl@ukr.de (S.B.); 2 Centre for Musculoskeletal Surgery, Department of Orthopaedics, Charité Universitaetsmedizin Berlin, Charitéplatz 1, 10117 Berlin, Germany

## 1. Comment

We read with great interest the article by Lunz et al. [1], in which the authors dealt with the new Periprosthetic Joint Infection (PJI)-TNM classification that was recently published by our group (Table 1) [2,3,4]. PJI represents one of the most feared complications in the orthopedic field, resulting in impaired quality of life, repeated and prolonged hospital stays, and significant morbidity and mortality in affected patients. Still, there is no commonly used classification system that could facilitate the comparison of treatment strategies and patient outcomes [5,6]. Therefore, we are delighted with the authors’ conclusions that “clinicians and researchers should be familiar with the new PJI-TNM classification and start implementing it into their routine practice” [1].

The work of Lunz et al. [1] retrospectively assessed 80 consecutive PJI patients treated with a two-stage exchange and was the first to correlate the PJI-TNM classification to surgical parameters and some clinical outcome parameters, such as need for revision surgery after stage one surgery, the duration of the interim period, and mortality. In addition, Lunz et al. [1] believed that the initial PJI-TNM publication from our group could be improved through certain modifications to the TNM backbone, resulting in a “pTNM” version. An additional “p-status” (type of prosthesis) was proposed to distinguish between standard implants (p0), revision implants (p1), and megaprostheses (p2). Further suggestions were to add an “x” in front of the “p-status” to indicate a loosened implant and to limit the criteria parameters for p, T, N, and M to only 0 = least serious, 1 = moderate, and 2 = most serious by eliminating the letters for the subclassifications of the 0, 1, and 2 categories of our initially proposed classification. They also proposed the replacement of the CCI for the assessment of patients’ comorbidities with the American Society of Anesthesiologists (ASA) Physical Status Classification System [7].

### 1.1. Correlation of the PJI-TNM Classification and Clinical Outcome

The interesting aspect of the publication of Lunz et al. [1] is the fact that the authors correlated the PJI-TNM classification to clinical outcome parameters based on eighty clinical cases with hip and knee PJI with two-stage treatment or the intention-to-treat in two stages. Outcome included surgical parameters of stage one surgery and clinical outcome parameters, such as need for revision surgery after stage one surgery, performed reimplantation, the duration of the interim period, and mortality. They found that prior history of septic revision of the same joint (“r-status”) showed a statistically significant correlation with a medium-sized effect with the type of spacer, bone loss, and the duration of stage one surgery. There was also a statistically significant association with a medium-sized effect between soft tissue and implant condition (“T-status”) and the probability of free implantation as well as blood loss and bone loss during the first stage of surgery. Furthermore, the comorbidity of the patient based on the Charlson comorbidity index (CCI) [8] was found to be statistically significantly associated with a medium-sized effect between morbidity of the patient (“M-status”) and mortality [1].

In general, we are delighted to see that our proposed initial classification [2,3] shows statistically significant correlation with clinical outcome parameters [1]. In our eyes, this is of upmost importance, particularly for the correlation of the “M-status” and mortality. This allows for the determination of the lethality risk for the patient with underlying PJI. This is of clinical interest as PJI can be considered a life-threatening disease with a 10-year mortality of up to 45%, comparable to or even higher than cancers, which have a mean 10-year mortality of up to 31% [9]. We are also happy to see that the “r-status” and the “T-status” show a significant correlation with surgical parameters and treatment outcome.

### 1.2. The Modified PJI-pTNM Classification

The authors assessed the same eighty clinical cases with the modified PJI-TNM classification and found statistical significance with a large effect between the type of infected prosthesis (“p-status”) and the type of spacer used for the interim period and bone loss during the first stage of surgery, with a medium-sized effect on bone loss during stage one surgery [1]. The new “T-status” (tissue condition) was associated with a medium effect with the probability for reimplantation, the type of spacer used for the interim period, the duration of the interim period, bone loss, and blood loss during the first stage of surgery. The assessment of the patient’s comorbidity using the ASA Physical Status Classification System was also associated with a statistically significant medium-sized effect with mortality. Also, the new “N-status” (non-human cells) showed statistically significant correlation with medium-sized effect with the rate of implant revision, the type of spacer used, the duration of interim period, and operating time at stage one surgery [1].

### 1.3. Comparison of the PJI-TNM Classification and the Modified PJI-pTNM Classification

We acknowledge the efforts of Lunz et al. to modify and simplify the originally proposed PJI-TNM classification as we also believe that this version was quite complex. But we also believe that PJI with its huge variety of clinical appearances is a complex disease that needs comprehensive classification to derive treatment guidelines and to enable accurate scientific assessment. We have recently developed a digital training app to familiarize colleagues with the principles and to facilitate initial use. We are also aware of the fact that classification should undergo further modifications, e.g., the TNM classification has been subject to multiple changes and adoptions over the last 40 years in oncology and its subdisciplines [10,11].

The introduction of the “p-status” proposed by Lunz et al. [1] shows significant correlation with four parameters for first-stage revisions surgery, and we applaud the proposal to classify megaprostheses separately, as the periprosthetic infection of megaprostheses is often associated with higher treatment efforts and worse outcomes [12]. However, the introduction of a fifth parameter also risks making the classification even more complex. Instead of implementing a fifth category with the p item, the T-category our initial version with the category “a” for a standard implant and “b” for a revision implant could be extended with “c” for megaprosthesis. This would maintain the idea of the initial version for category “T” to cover the entirety of the tissue and the implant status.

The modified “T-status” and “N-status“ of Lunz et al. [1] resulted in more statistically significant correlations with surgical parameters for first-stage surgery compared to our initial version. Regarding the T-status, the addition of the presence of a fistula to the T2 together with a significant soft tissue defect seems problematic in our eyes. Fistulae or sinus tracts are common in PJIs and can be treated with simple excision during a two- or even a one-stage procedure [13]. On the other side, severe soft tissue defects are normally harder to treat, may lead to the polymicrobial colonization of the indwelling prosthesis, and frequently require free flap surgery with an overall worse outcome compared to standard wound closure procedures [14,15,16] and, in our eyes, should not be put into the same category as fistulae.

The modification of the N-parameter to N0: immature biofilm; N1: mature biofilm and “non-difficult-to-treat” bacteria; and N2: mature biofilm with “non-difficult-to-treat” bacteria resulted in four significant correlations with intraoperative criteria for stage one surgery (duration and type of spacer), the need for revision of the spacer, and the duration of the interim period. The initially presented N-category did not show any statistical correlation. Therefore, the modification of the N-criterion seems interesting for the future.

A major feature of the modifications made by Lunz et al. is the replacement of the CCI with the ASA classification system for the assessment of the “M-status” of the patient. The use of the CCI also showed a significant correlation with mortality. However, the ASA Physical Status Classification System is only associated with a *p*-value of 0.04 (significant correlation) versus a *p*-value of <0.01 (highly significant correlation) of the CCI with mortality. The CCI has shown good correlation with outcomes in musculoskeletal infections and orthopaedic surgery [17]. We agree that the determination of CCI requires more effort than the ASA score. However, we have recently introduced a learning PJI-TNM educational app that is available on Google Play (https://play.google.com/store/apps/details?id=de.ukr.pjitnm&pli=1, accessed on 30 July 2023) and the Apple Store (https://apps.apple.com/de/app/pji-tnm-educational-app/id1616664776, accessed on 30 July 2023) to facilitate the generation of the CCI based on the underlying diseases of the patient. Therefore, we would prefer to remain with our initial version, utilizing the CCI.

### 1.4. Future Perspectives

Future studies should focus on the correlation of the PJI-TNM classification and other important outcome values, such as reinfection rate, need for surgical revision after stage two surgery, or the quality of life of the patient, which has not yet been studied. Furthermore, the success of other treatment approaches, such as DAIR (debridement, antibiotics, and implant retention) or one-stage exchange, should be evaluated based on the PJI-TNM classification. Finally, prospective multicenter studies are necessary to determine the success rates of existing treatment concepts and to standardize treatment strategies, as already successfully practiced in oncology.

In conclusion, the PJI-TNM classification, as both the initial and the modified version, has shown its correlation with important surgical and outcome parameters and is of value for the classification of PJI in the future. We will continue to work on the validation and correlation of clinical parameters with the PJI-TNM classification in order to improve classification, treatment, outcome, and scientific assessment of PJIs in the future.

## Figures and Tables

**Table 1:** The PJI-TNM classification as introduced by Alt et al. [2,3].

PJI-TNM Classification	
r—reinfection	If the infection involves a previously infected implant, the situation is considered to be “reinfection” and an “r” is placed in front of the classification
T—tissue and implant conditions	
T0a	Stable standard implant without important soft tissue defect
T0b	Stable revision implant without important soft tissue defect
T1a	Loosened standard implant without important soft tissue defect
T1b	Loosened revision implant without important soft tissue defect
T2a	Severe soft tissue defect with standard implant
T2b	Severe soft tissue defect with revision implant
N—non-human cells	
N0a	No mature biofilm formation (former: acute), directly postoperatively
N0b	No mature biofilm formation (former: acute), late haematogenous
N1a	Mature biofilm formation (former: chronic) without “difficult to treat bacteria”
N1b	Mature biofilm formation (former: chronic) with culture negative infection
N2a	Mature biofilm formation (former: chronic) with “difficult to treat bacteria”
N2b	Mature biofilm formation (former: chronic) with polymicrobial infection
N2c	Mature biofilm formation (former: chronic) with fungi
M—morbidity of the patient	
M0	Not or only mildly compromised (Charlson comorbidity index: 0–1)
M1	Moderately compromised patient (Charlson comorbidity index: 2–3)
M2	Severely compromised patient (Charlson comorbidity index 4–5)
M3a	Patient refuses surgical treatment
M3b	Patient does not benefit from surgical treatment
M3c	Patient does not survive surgical treatment
